# Expressed sequence tags from *Peromyscus *testis and placenta tissue: Analysis, annotation, and utility for mapping

**DOI:** 10.1186/1471-2164-9-300

**Published:** 2008-06-24

**Authors:** Julie L Weston Glenn, Chin-Fu Chen, Adrienne Lewandowski, Chun-Huai Cheng, Clifton M Ramsdell, Rebecca Bullard-Dillard, Jianguo Chen, Michael J Dewey, Travis C Glenn

**Affiliations:** 1 *Peromyscus *Genetic Stock Center, Department of Biological Sciences, University of South Carolina, Columbia, SC 29208, USA; 2 Department of Genetics and Biochemistry, Clemson University, and Clemson University Genomics Institute, Clemson, SC 29634, USA; 3 Department of Genetics and The Carolina Center for Genome Sciences, University of North Carolina, Chapel Hill, NC 27599, USA; 4 Department of Biology, Claflin University, Orangeburg, SC 29115, USA; 5 Savannah River Ecology Laboratory, Aiken, SC 29803, USA; 6 Department of Environmental Health Science, University of Georgia, Athens, GA 30602, USA

## Abstract

**Background:**

Mice of the genus *Peromyscus *are found in nearly every habitat from Alaska to Central America and from the Atlantic to the Pacific. They provide an evolutionary outgroup to the *Mus/Rattus *lineage and serve as an intermediary between that lineage and humans. Although *Peromyscus *has been studied extensively under both field and laboratory conditions, research has been limited by the lack of molecular resources. Genes associated with reproduction typically evolve rapidly and thus are excellent sources of evolutionary information. In this study we describe the generation of two cDNA libraries, one from placenta and one from testis, characterize the resulting ESTs, and describe their utility for mapping the *Peromyscus *genome.

**Results:**

The 5' ends of 1,510 placenta and 4,798 testis clones were sequenced. Low quality sequences were removed and after clustering and contig assembly, 904 unique placenta and 2,002 unique testis sequences remained. Average lengths of placenta and testis ESTs were 711 bp and 826 bp, respectively. Approximately 82% of all ESTs were identified using the BLASTX algorithm to *Mus *and *Rattus*, and 34 – 54% of all ESTs could be assigned to a biological process gene ontology category in either *Mus *or *Rattus*. Because the *Peromyscus *genome organization resembles the *Rattus *genome more closely than *Mus *we examined the distribution of the *Peromyscus *ESTs across the rat genome finding markers on all rat chromosomes except the Y. Approximately 40% of all ESTs were specific to only one location in the *Mus *genome and spanned introns of an appropriate size for sequencing and SNP detection. Of the primers that were tried 54% provided useful assays for genotyping on interspecific backcross and whole-genome radiation hybrid cell panels.

**Conclusion:**

The 2,906 *Peromyscus *placenta and testis ESTs described here significantly expands the molecular resources available for the genus. These ESTs allow for specific PCR amplification and broad coverage across the genome, creating an excellent genetic marker resource for the generation of a medium-density genomic map. Thus, this resource will significantly aid research of a genus that is uniquely well-suited to both laboratory and field research.

## Background

Members of the genus *Peromyscus *are mice found from Alaska to Central America and from the Atlantic to the Pacific. They occur in a wide range of habitats including sea-level wetlands and beaches, forests, prairies, deserts, and mountains of elevation up to 14,000 ft. This genus contains not only the two most wide-spread mammals in North America, the deer mouse (*P. maniculatus*) and the white-footed mouse (*P. leucopus*), but also contains North America's most endangered mammal, the Perdido Key Beach Mouse (*P. polionotus trissyllepsis*).

Peromyscines are unique non-traditional research models and have been studied extensively under both field and laboratory conditions in such diverse areas as epidemiology, speciation, habitat adaptation, behavior, toxicology, and aging [1-9]. Several strains maintained at the *Peromyscus *Genetic Stock Center (PGSC) exhibit neurological defects and stereotypical behavior that are not characterized in either *Mus *or *Rattus*. Furthermore, they are the reservoirs of several emerging human diseases, including hanta virus pulmonary syndrome [10,11], lyme disease [12], ehrlichiosis, and babesiosis [11].

**Figure 1:**
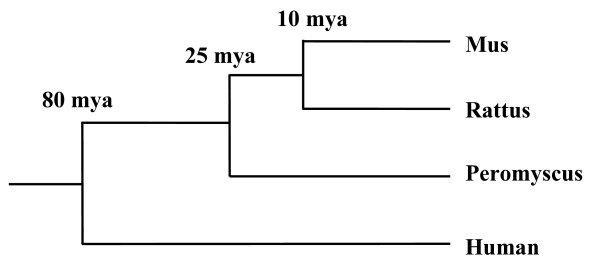
**Phylogenetic relationships of *Peromyscus *to *Mus*, *Rattus*, and Human**. Divergence dates are estimated from [13,38].

Although *Peromyscus *species are phenotypically similar to *Mus *and *Rattus*, *Peromyscus *is an exclusively North American genus and is only distantly related to these Old World species, having diverged from the common ancestor of the *Mus/Rattus *lineage ca. 25 mya [13] (Fig. 1). Thus, they not only provide an excellent outgroup for evolutionary study of *Mus *and *Rattus*, they also provide an additional group for evolutionary studies between these two common laboratory models and humans. Like *Mus *and *Rattus*, *Peromyscus *are readily adaptable to laboratory conditions. However, their natural variation makes them better suited for modeling the effects of genetic diversity on a trait of interest.

Despite the abundance of studies on this genus, research has been limited by the lack of molecular genetic resources. Recently though, there have been significant contributions in this area in the form of BAC libraries for *P. maniculatus rufinus *(Childrens' Hospital Oakland Research Institute, CHORI-233) and *P. leucopus *(J. Storz, Univ. Nebraska), hundreds of microsatellite loci [[14,15], Hoekstra and Glenn personal communication], and interspecific backcross and whole-genome radiation hybrid cell mapping panels [16,17]. A genomic linkage map of *Peromyscus *is needed to fully exploit all of these resources and advance *Peromyscus *as a model species.

To aid in the production of a linkage map, cDNA libraries of placenta tissue and testis tissue were constructed and used in the production of type I (gene-coding) markers. Placenta and testis were chosen because genes regulating reproduction are known to evolve rapidly [18,19]. Therefore, genes expressed in these tissues are likely to contain polymorphisms that are easily detectable in interspecific backcross and radiation hybrid mapping panels. By sequencing clones from cDNA libraries the expressed portion of the genome can be characterized. These expressed sequence tags (ESTs) may then be identified by homology to *Mus *and *Rattus*, thus providing data useful for evolutionary analysis, gene expression, and mapping. Below we characterize sequences of clones from these cDNA libraries.

## Results and Discussion

### Overview

We sequenced the 5' ends of 1,510 placenta clones and 4,798 testis clones. After removing low quality sequences, clustering sequences into gene families, and contig assembly, there remained 904 unique placenta and 2,002 unique testis sequences (Table 1). We then determined the number of EST sequences that typically constituted a cluster (Table 2). At the clustering stage, 87.4% (785/898) of the placenta ESTs belong to unique singletons because they did not share > 100 bp identity with any other EST. For the testis library, 90.8% (1,803/1,985) of the testis ESTs were singletons. After related ESTs were clustered into gene families, ESTs within those clusters were assembled into contigs representing unique genes. For the placenta library, this resulted in 893 clusters containing just one long sequence, while three clusters contained two contigs and one cluster contained 5 contigs. For the testis library, 1,993 clusters contained a single contiged sequence and one contained > 5 contigs.

**Table 1:** Number of ESTs at each stage of the analysis

Stage	Placenta	Testis
		
	Number of Sequences	Number of Sequences
Initial input	1510	4798
Quality analysis	1358 (89.9%)	3917 (81.6%)
Vector trimming	1135 (75.2%)	2695 (56.2%)
Clustering and Contig assembling	904 (59.9%)	2002 (41.7%)

**Table 2:** Number of clusters of different sizes after the cluster and assemble stages of the TGICL algorithm

Size*	Placenta		Testis	
		
	Cluster stage	Assemble stage	Cluster stage	Assemble stage
1	785	893	1803	1993
2	65	3	129	0
3	23	0	22	0
4	9	0	15	0
5	8	1	6	0
>5	8	0	10	1

*Refers to the number of EST sequences in a cluster at the clustering stage and the number of contigs at the assemble stage.

**Figure 2:**
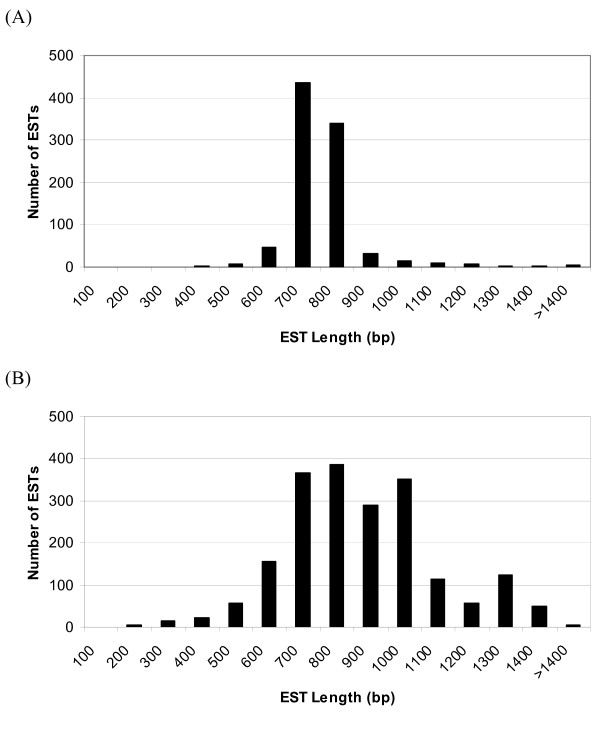
**Distribution of EST lengths**. Distribution of (A) placenta and (B) testis EST lengths.

To analyze for any size bias in this collection of ESTs, we examined the distribution of EST lengths as well as their average. For placenta, EST length ranged from 139 – 2,777 bp with an average of 711 bp (Fig. 2A). Over 85% were between 700 – 900 bp, which is consistent with the aforementioned finding that 87.4% of placenta clusters contained only a single EST and that the maximum reads with our sequencers is ~900bp. For testis, the range of EST lengths was 136 – 2,424 bp with an average length of 826 bp (Fig. 2B). These average lengths are likely limited by sequencing technology. Therefore, most of the genes represented in these libraries are likely much longer. All ESTs have been deposited in GenBank with continuous accession numbers of EV468245 – EV472065.

### EST annotation and function

Analysis of the *Peromyscus *EST sequences using the BLASTX algorithm resulted in the identification of 2,377 *Mus musculus *and 2,385 *Rattus norvegicus *orthologs, nearly all of which yielded identical results (Table 3). Thus, approximately 82% of all ESTs were identified. Inability to identify the remaining 18% could be due to a multitude of causes, including but not limited to: significant sequence divergence of *Peromyscus *genes from *Mus *and *Rattus*, genes unique to *Peromyscus*, or sequences from untranslated regions, which would not be identified in a BLASTX search because BLASTX only compares translated amino acid sequences. Although BLASTN searches may reveal additional orthologs, we did not perform them on unidentified ESTs in order to remain as conservative as possible.

**Table 3:** Summary of BLASTX results.

	Placenta		Testis	
	(EST input = 904)		(EST input = 2002)	
	mouse	rat	mouse	rat
Total hits*	882	884	1935	1947
Non-redundant hits^†^	781	775	1596	1610

EST contig sequences were BLASTed against mouse (*Mus*) and rat (*Rattus*) Refseq database using BLASTX. Only matches with e-values of ≤ 10 were considered significant.

*The homolog hit with lowest e-value was selected (when multiple hits existed).

^†^Redundant homolog records were removed when more than one EST hit with the same homolog.

**Figure 3:**
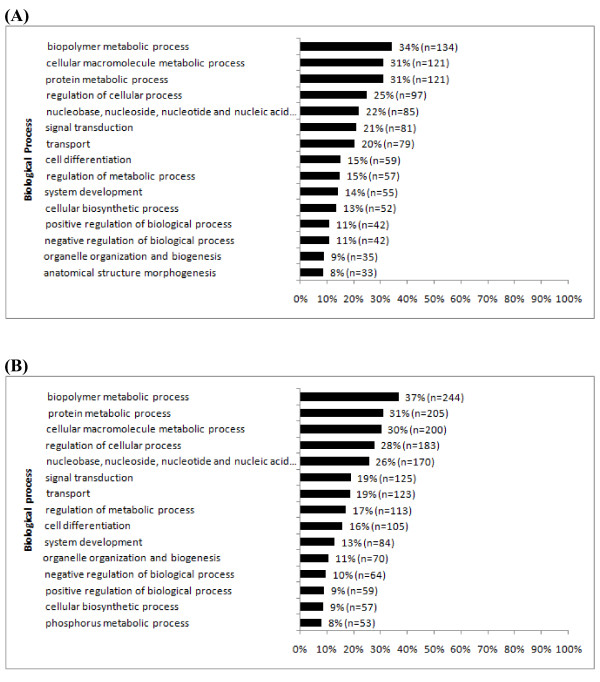
**Gene ontology categories**. Top 15 gene ontology categories for biological processes of (A) placenta and (B) testis ESTs as annotated using *Rattus *homologs. GO terms were obtained using the online tool, FatiGO [34]. The 'n' denotes the number of genes containing the same GO term, and the percentage represents the ratio of the number of genes annotated with the same GO term versus the total number of genes with GO annotation (some genes do not have GO information and many genes have multiple GO annotations).

Once the ESTs were identified they were further classified according to their biological processes or gene ontology. Of 904 placenta ESTs, 492 (54.4%) and 399 (44.1%) had a known biological process term associated with their function in *Mus *and *Rattus*, respectively. For the 2,002 testis ESTs these numbers were 835 (41.7%) and 674 (33.7%) for *Mus *and *Rattus*, respectively. Examination of the 15 most common gene ontology (GO) categories for *Rattus *orthologs of the placenta ESTs indicated that they are primarily involved in multiple metabolic processes, transport, and signal transduction (Fig. 3A). For the mouse orthologs, the top 15 categories are the same and occur in approximately the same proportions. Differences were very minor and likely due to more complete annotation of the *Mus *genome. For the testis ESTs, the *Rattus *orthologs' biological processes are very similar to the placental ESTs. They are primarily involved in multiple metabolic processes, transport, and signal transduction (Fig. 3B) and the *Mus *orthologs again have nearly identical biological processes.

### Utility of ESTs for mapping

**Figure 4:**
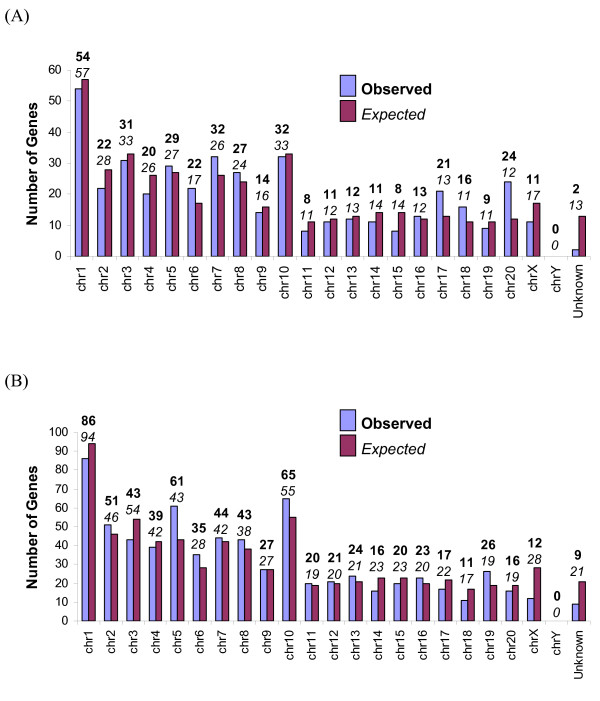
**Chromosome distribution**. Chromosome distribution for (A) 429 rat placenta homologes and (B) 709 rat testis homologes.

Because the *Peromyscus *genome organization is known to be more similar to *Rattus *than *Mus *[16,20], the distribution of ESTs across the rat genome is likely to be representative of the distribution in *Peromyscus*. To determine if these libraries represent genes from all chromosomes proportionally, the numbers of annotated ESTs occurring on the autosomes and the X chromosome in the *Rattus *genome were compared to the numbers of ESTs that would be expected to occur on each chromosome. Expected numbers for each chromosome were based on the proportion of total *Rattus *genes represented on each chromosome, and those proportions then scaled to a library of the same size as the placenta and testis libraries. Thus, chromosomes whose genes are over- or under-represented in the libraries may indicate areas of abundant or reduced transcription, respectively. The observed EST distribution in fact does differ significantly from the expected random distribution for both placenta and testis (χ^2 ^= 32.56, df = 20, *P *= 0.023 and χ^2 ^= 34.49, df = 20, *P *= 0.038, respectively; Fig. 4). Because of the large sample sizes (N = 427 for placenta, N = 700 for testis) the chi-square results may reflect only minor differences, as the observed and expected numbers rarely differed by more than a few ESTs. However, as functional groups are frequently found on the same chromosome resulting in linkage disequilibrium [21,22], the result is not surprising. Here, the difference between expected and observed as a proportion of the expected number of ESTs is potentially informative. For example, in the placenta library, 100% more ESTs were observed on *Rattus *chromosome 20 than would be expected by chance alone (e.g., expected = 12, observed = 24). Similarly, *Rattus *chromosomes 17 and 18 had 62% and 46% more ESTs, respectively, than would be expected from random expectation. Thus, the abundance of the genes identified from these three chromosomes may reflect the importance for the development and maintenance of placental tissue and also reflect specific placental functions (e.g., endocrine activities, transport, and/or intrauterine invasion).

Conversely, rat chromosomes 15 and X had 43% and 35% fewer genes than expected. Under-representation of placental genes on the rat X chromosome is surprising, as the *Mus *X chromosome is known to be enriched for genes primarily transcribed only in female mice [23], an observation attributed to the silencing of the paternal X chromosome in the placenta of female mammals [24]. This paucity of placental genes cannot be explained by evolutionary differences among *Mus*, *Rattus*, and *Peromyscus*, as fluorescent in situ hybridization shows the genes on the X chromosome are shared by all three species [20]. Thus, these female-specific transcripts on the X chromosome may have little to do with placentation. Alternatively, placentation genes on the X chromosome may have diverged significantly from *Mus *and *Rattus*, thus reducing the likelihood of positive identification with the BLAST search.

For the testis library, an abundance of testis-related genes were found on rat chromosomes 5 and 19, which had 42% and 37% more genes than random expectation. These chromosomes likely account for the paucity of testis-related genes on rat chromosomes 14, 18, and X, which have 30%, 35%, and 57% fewer genes than expected by chance. The lack of testis-related genes on the X chromosome is not surprising, as recent research demonstrates the X chromosome lacks genes involved in spermatogenesis [23,24]. The only chromosomes that are consistently under-represented in both libraries are the X and Y. In fact, if the X chromosome is excluded, the observed distribution does not differ from expected for either the placenta or testis library (χ^2 ^= 29.25, df = 19, *P *= 0.062 and χ^2 ^= 24.90, df = 19, *P *= 0.164, respectively), although this result is only marginal for the placenta library. For all other chromosomes, these two libraries provide a sufficient number and distribution of markers to effectively span at least 90% of any given chromosome, therefore providing a valuable and effective marker resource for mapping the *Peromyscus *genome. In fact, prior to the development of these ESTs, the *Peromyscus *Genetic Stock Center had mapped most of only 2 chromosomes. Those 2 chromosomes were finished with the aid of new EST markers, and all or parts of 11 additional new chromosomes were mapped using mostly the EST markers [16]. Recently, 4 additional chromosomes have been partially mapped using only EST markers (unpublished data).

Although abundant, the most useful ESTs are those located in unmapped regions. Of 377 ESTs occurring in areas that would bridge gaps in the existing *Peromyscus *genome map, a BLASTN search indicated that 2 matched non-coding DNA better than they matched their associated protein, 4 yielded no significant similarity, and 5 matched a different chromosome than expected. These few unidentified ESTs may have resulted from a failure of the BLASTN algorithm to recognize more highly diverged sequences as orthologous. Of the remaining 366 ESTs, 151 (41.3%) ESTs offered good candidates for primer design, of which 81 (22.8%) were selected for initial testing. To qualify as a good candidate for primer design, a BLASTN of the EST sequence to *Mus *must match the expected protein only and either span an intron 300 – 1000 bp or ≥ 500 bp of the EST must occur in an untranslated region. Of the 81 primer pairs designed and tested, 44 (54.3%) were easily amplified and contained polymorphisms useful as assays for typing on a backcross panel of *Peromyscus maniculatus *× *P. polionotus *(see methods). This number is a conservative estimate, however, as 22 (27.2%) have been optimized but not sequenced because they were not needed.

Of 904 placenta ESTs with a significant protein match based on a BLASTX search, 657 also had a single high-probability BLASTN match to the *Mus *genome, and therefore were likely to be highly specific for mapping purposes. Of these, 29 ESTs (4.4%) contained 34 microsatellite repeats distributed as 7, 18, 8, and 1 di-, tri-, tetra-, and pentanucleotide microsatellites. One EST contained 2 trinucleotide microsatellites, 1 contained 3 trinucleotide microsatellites, and 2 contained a di- and a tetranucleotide microsatellite. For 1409 testis ESTs with only one BLASTN match to the *Mus *genome, 111 sequences (7.9%) had 134 microsatellite repeats distributed as 35, 73, 20, 4, and 2 di-, tri-, tetra-, penta-, and hexanucleotide microsatellites. Three contained 2 dinucleotide microsatellites, 6 had 2 trinucleotide repeats, 2 had 3 trinucleotide repeats, and 4 contained 2 tetranucleotide repeats. In addition, one EST each contained a di- and a tri-, a di- and 3 tri-, a di- and tetra-, and a tetra- and pentanucleotide microsatellite. Because of the high variability of microsatellites within a population, they are ideally suited for QTL analysis. Thus, these libraries not only serve as markers for general map construction but as markers that will allow the discovery of genes underlying phenotypic variation. Microsatellites found in these libraries may be particularly useful in this regard because they are actually contained within known genes. This is a distinct advantage over most microsatellites which are found in anonymous, non-coding regions and are associated with specific proteins only by virtue of physical proximity.

## Conclusion

The generation of several thousand ESTs from reproductive tissues has significantly expanded the molecular resources available for the genus *Peromyscus*. This provides an invaluable resource of genetic markers for constructing genomic linkage maps of the genus, a project currently underway and partially completed by the *Peromyscus *Genetic Stock Center and others [[16,17], Hoekstra personal communication]. The resulting map will better enable researchers to genetically examine phenotypes in a species displaying naturally-occurring genome variation. A *Peromyscus *linkage map will also provide information for studying the evolution of rodent genome organization, in particular by aiding in the reconstruction of the ancestral rodent genome. Such evolutionary insight on the functional organization of the rodent and mammalian genomes may help link abundant *Mus *and *Rattus *research to human studies. In addition, these ESTs provide a resource for informative microarray and QTL analyses and single nucleotide polymorphism discovery. These uses will be particularly informative in *Peromyscus *because several species are known to hybridize in the laboratory. Identification of the genetic differences between interbreeding species can further our understanding of hybrid dysgenesis and genomic imprinting [2,25,26]. Thus, the development of these libraries will allow *Peromyscus *research to answer questions that traditional *Mus *and *Rattus *models simply cannot address.

## Methods

### Library construction and EST isolation

Testis tissue was taken from a 6-month old sexually mature virgin male *Peromyscus maniculatus bairdii *and placed immediately into TRIZOL^® ^reagent (Invitrogen Corporation). Placentas were collected from three *Peromyscus maniculatus bairdii *(BW), two *P. polionotus subgriseus *(PO), and one placenta derived from a hybridization between two subspecies, PO and *P. p. leucocephalus *(LS). Because *Mus *placentas are considered to reach maximum size and maturity at e16.5 [27] all placentas were collected at e17 – 18 except one BW collected at e16. Placentas were mixed because *P. maniculatus *and *P. polionotus *are sister species able to interbreed and were used in several mapping panels. Thus, a library representing maximum diversity was highly desirable. Diversity was enhanced further by the inclusion of maternal decidual tissue which may be under selective pressure similar to the fetus' portion of the placenta. Results from the mapping panels indicate differences are typically single nucleotide polymorphisms which do not interfere with primer optimization and gene amplification [5,16]. Testis RNA was sent to Amplicon Express (Pullman, WA) for cDNA library construction and placenta RNA was sent to Stratagene (La Jolla, CA). Both libraries were produced in lambda bacteriophage using the Uni-ZAP^® ^XR vector (Stratagene, La Jolla, CA). The libraries were amplified but not normalized. Inserts were excised according to the mass excision protocol described in the Stratagene manual [28] and the resulting phagemids were transfected into SOLR™ *Echerichia coli *cells and plated on LB-Ampicillin (0.1 mg/ml) agar plates. Cells were grown in a 37°C incubator for 15 – 20 hours. Colonies were picked into 300 μl of LB-Ampicillin (0.05 mg/ml) broth in a deep-well plate and grown in a 37°C incubator with shaking overnight.

Inserts were amplified in 10 μl Polymerase Chain Reactions (PCRs) with the following concentrations: 1× PCR buffer, 1.5 mM MgCl_2_, 25 μg/ml Bovine Serum Albumin, 0.2 mM dNTPs, 0.4 mM forward primer, 0.4 mM reverse primer, 0.05 units Taq, and 1 μl of amplified bacteria colony. Thermal cycler conditions were: initial denaturation at 94° for 3 minutes, followed by 10 cycles of (94°C for 20 seconds, 50°C for 20 seconds, 72°C for 3 minutes 30 seconds), cycles 11 – 30 added 10 seconds/cycle to the 72°C extension, and ended at a 15°C hold.

Presence of inserts was verified on a 1% agarose gel. Colonies containing inserts were identified and their PCR products purified by combining 4 μl PCR product, 5 units Exonuclease I, and 0.75 units Shrimp Alkaline Phosphatase, and incubating at 37°C for 15 minutes, 80°C for 15 minutes, then holding at 15°C. Samples were sequenced from the 5' end using 2.0 μl purified PCR product plus 0.75 μL BigDye v3.1, 1.75 μL 5× Sequencing Dilution Buffer, 1.25 μL T3 Primer (3.3 μM), and 4.25 μL H_2_O. Cycling conditions were 70 cycles of 96°C for 10 seconds, 50°C for 5 seconds, and 60°C for 4 minutes, ending with a final hold at 15°C.

Sequencing reactions were precipitated by adding 1 μl of 1.5 M NaOAc + 250 mM EDTA, then 40 μl of cold 95% ethanol, mixing, and placing on ice for 15 minutes. Samples were centrifuged at 1,500 × G for 45 minutes and the ethanol removed. Pellets were resuspended in Hi-Di and run on an ABI capillary sequencer (either a 3100-Avant, 3130 XL, or a 3730 XL; Applied Biosystems, Foster City, CA).

### EST processing

We sequenced a total of 7,387 ESTs. Removing redundant files resulted in 1,510 placenta and 4,798 testis sequences. The initial processing consisted of two steps: (1) quality control and vector cleaning, and (2) sequence clustering and contig assembling. The sequences with Phred quality values [29] lower than 25 were first removed from further analysis. Vector cleaning was performed using the Phrap/Cross_match/Swat software [30]. Sequences contaminated with pBluescript vector or *E. coli *gene sequences were removed as were sequences with fewer than 100 good bases (i.e., quality value < 25) and sequences with more than 5% ambiguous bases (i.e., 'N').

We then used the TIGR gene index procedure (i.e. TGICL algorithm) [31] to cluster raw EST sequences into groups of highly related sequences, possibly a family of genes, and then to assemble those sequences into contigs consisting of the longest non-redundant stretch of the multiply aligned ESTs (program CAP3, included in TGICL). These contigs are likely to represent individual genes. Unlike NCBI's UniGene procedure which only gathers similar ESTs together, the TIGR gene index procedure allows clustering of ESTs based on a pre-selected criterion (base pair identity in this case). We specified that ESTs with ≥ 100 bp identity should be put together in the same cluster. However, multiple contigs within a cluster were possible if there was no way to assemble all similar sequences into a single contig.

### EST annotation and function

We utilized the BLASTX procedure for the translated protein-protein comparison with both the *Mus musculus *and *Rattus norvegicus *Refseq databases to identify homologs. We also performed BLASTN on a limited number of ESTs and the results were identical. BLASTN was used because some EST sequences may have contained only untranslated regions and therefore would not have shown up on BLASTX searches. Only matches with an e-value ≤ 10 were considered significant.

A Chi-square goodness-of-fit test (Proc FREQ) [32] was used to determine if the number of ESTs on each rat chromosome was equal to the number expected. Expected numbers of proteins for each *Rattus *chromosome were taken from a count of protein accession numbers from the rat protein RefSeq database [33]. By this count, the *Rattus *genome contains 34,738 proteins with known chromosomal locations. By calculating the proportion of genes on each chromosome we were able to determine expected frequencies by multiplying that proportion by the total number of ESTs with known chromosomal locations for each library (N = 427 for placenta, N = 700 for testis). Probability was assigned on the basis of a 1-tailed test at *P *≤ 0.05.

To determine what biological processes were associated with the identified *Peromycsus *ESTs, we analyzed the mouse and rat homologous gene lists using an online gene ontology analysis tool, FatiGO [34]. We assigned gene ontologies using the fourth level of increasing specificity.

### Primer design and use of ESTs in mapping

Because of the similarity of the *Peromyscus *genome to the rat genome, we identified regions of the rat genome for which we wanted markers, spacing markers ca. 15 – 20 Mb apart. Based on BLASTX results, we identified ESTs in those regions and re-BLASTed to the *Mus *genome (NCBI Build 36) using the Map Viewer option on the National Center for Biotechnology Information website [33]. We chose to BLAST to *Mus *instead of *Rattus *because of the greater abundance of annotated *Mus *sequence. We also used regular megablast instead of the cross-species megablast to be conservative in our gene assignments. The Map Viewer option allowed us 1) to identify which ESTs matched the expected protein only, thus limiting non-specificity of primers, and 2) to easily identify locations and sizes of introns.

Choosing good candidates for primer design was further limited to those ESTs which spanned a 300 – 1000 bp intron in *Mus *or for which ≥ 500 bp of the EST occurred in an untranslated region. Because introns and untranslated regions are not always well-conserved across species, these criteria maximized the possibility of amplifying a PCR fragment small enough to be sequenced from both ends with overlap, but large enough to increase the likelihood of finding interspecific single nucleotide polymorphisms (SNPs) which could be exploited in an interspecific backcross panel. Once these regions were identified, we designed primers using Oligo 6.0 (Molecular Biology Insights, Inc.).

The mapping panel was made from offspring of *P. maniculatus *(BW) × *P. polionotus *(PO) F1 hybrid males backcrossed to BW females. The panel was comprised of four unrelated families, three of which contributed 22 offspring each to the panel and the fourth contributed 20 offspring for a total panel size of 86. All primers were optimized using BW and PO DNA from animals unrelated to the mapping panel using touchdown (TD) protocols (either TD65, TD60, or TD55) [35]. Then, a BW and a PO PCR product were cleaned and sequenced in both forward and reverse directions in the same manner as described for "library construction and EST isolation" above. Sequences were aligned in Sequencher (GeneCodes Corporation) and SNPs identified. Because all individuals in the backcross panel had at least one BW allele we used the SNP-RFLPing program [36] to search for enzymes that would exploit a SNP and cut only the PO allele. PCR fragments were then amplified from the backcross panel, digested, and scored on either 2% agarose or 5% acrylamide.

All ESTs with only a single BLASTN match to the *Mus *genome, and therefore specific enough to be markers useful for mapping, were screened for simple sequence repeats (SSRs) using msatCommander 0.8.1 [37]. This script searches for repeats with the lowest alphabetical designation that are unique and non-complementary. ESTs from both libraries were searched for di-, tri-, tetra-, penta-, and hexanucleotide SSRs. A minimum of 4 repeat units was required for all except dinucleotide SSRs for which a minimum of 6 repeats was specified.

## Authors' contributions

MJD conceived of the project, obtained libraries and generated the backcross panel. TCG was responsible for the laboratory protocols. AL and JLWG isolated and prepared ESTs for sequencing, while TCG ran sequences with help from JC and RB–D. C–FC constructed the pipeline to analyze and annotate all sequences with significant aid from C–HC. CMR, JLWG, and AL designed primers which CMR and AL screened on the backcross panel. JLWG and C–FC drafted the manuscript. All authors read and approved the manuscript.
